# Continuous Nanoprecipitation of Polycaprolactone in Additively Manufactured Micromixers

**DOI:** 10.3390/polym14081509

**Published:** 2022-04-07

**Authors:** Simeon Göttert, Irina Salomatov, Stephan Eder, Bernhard C. Seyfang, Diana C. Sotelo, Johann F. Osma, Clemens K. Weiss

**Affiliations:** 1 Technische Hochschule Bingen, Life Sciences and Engineering, Berlinstrasse 109, 55411 Bingen, Germany; simeon.goettert@stud.th-bingen.de (S.G.); irina.salomatov@web.de (I.S.); s.eder@th-bingen.de (S.E.); b.seyfang@th-bingen.de (B.C.S.); 2 Department of Electrical and Electronic Engineering, Universidad de los Andes, Cra. 1E No. 19A-40, Bogotá 111711, Colombia; dc.sotelo10@uniandes.edu.co (D.C.S.); jf.osma43@uniandes.edu.co (J.F.O.)

**Keywords:** ouzo effect, nanoparticles, continuous process, polycaprolactone, 3D printing, micromixer

## Abstract

The polymeric ouzo effect is an energy-efficient and robust method to create nanoparticles with biologically degradable polymers. Usually, a discontinuous or semi-continuous process is employed due to its low technical effort and the fact that the amount of dispersions needed in a laboratory is relatively small. However, the number of particles produced in this method is not enough to make this process economically feasible. Therefore, it is necessary to improve the productivity of the process and create a controllable and robust continuous process with the potential to control parameters, such as the particle size or surface properties. In this study, nanoparticles were formulated from polycaprolactone (PCL) in a continuous process using additively manufactured micromixers. The main goal was to be able to exert control on the particle parameters in terms of size and zeta potential. The results showed that particle size could be adjusted in the range of 130 to 465 nm by using different flow rates of the organic and aqueous phase and varying concentrations of PCL dissolved in the organic phase. Particle surface charge was successfully shifted from a slightly negative potential of −14.1 mV to a negative, positive, or neutral value applying the appropriate surfactant. In summary, a continuous process of nanoprecipitation not only improves the cost of the method, but furthermore increases the control over the particle’s parameters.

## 1. Introduction

**Figure 1:**
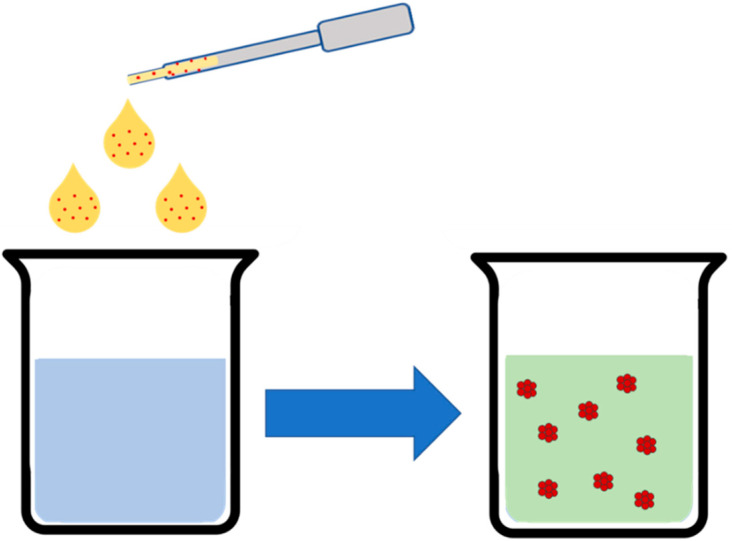
Schematic representation of the “ouzo effect”. The organic phase containing the polymer is transferred to the aqueous phase. The polymer then aggregates due to lower solubility in the mixture.

During the last decades, research for carriers of medicinal substances and supplements has increasingly been given attention [1,2]. This attention can be traced back to the desire for improving the solubility, stability and biodistribution of the active ingredients [2,3,4,5,6,7,8]. Amidst the various attempts to produce carriers, one method is known for its reproducibility and its simple implementation [1,9,10]. The process is called the “Ouzo-effect” [9,10,11]. With this method, it is possible to create polymer carriers, better known as nanoparticles [4]. Those nanoparticles, which usually have a size of 100–200 nm [5], shall act as a shell for those medicinal substances to protect them from degradation and to improve cellular uptake [7,12,13]. The ouzo effect has its origins in the Greek alcoholic spirit, which is the template of this encapsulation. Ouzo is made of 55 v% water, 45 v% ethanol and 0.2 v% anethol [9]. When ouzo is diluted with water, phase separation of the anethol occurs as its reduced solubility in the resulting mixture. The anethol begins to form droplets that can be seen in the change of the liquid from having a clear appearance to a milky one [11,14]. This process was described to be a suitable method for particle formation when a polymeric component is used instead of anethole (“polymeric Ouzo-effect”) [9]. The process is schematically shown in Figure 1. In contrast to emulsion heterophase polymerization processes, little energy input is required [1,15]. Several other processes, referred to as nanoprecipitation, solvent diffusion or solvent displacement [10], describe the spontaneous formation of nanoparticles via a nucleation-and-growth or a nucleation-and-aggregation mechanism [16,17]. Due to the similarities in particle formation mechanisms, however, Mamusa et al. justifies summarizing all of these processes as “Ouzo effect” [10].

In the meantime, many studies have been performed trying to use the ouzo effect to create proper nanoparticles that can be tested in cellular uptake studies [5]. The set up in those studies is to dissolve the polymer in a water miscible organic solvent and mix it with an aqueous phase [5,13,18]. Most organic phases consist of solvent, easy to evaporate after the formulation, such as acetone or tetrahydrofurane (THF) [1,7,13]. The aqueous phase usually consists of water, in some cases with surfactants as an additive [13]. Those surfactants are used to improve the colloidal stability of the particles [9,13], but can also influence the particles’ surface charge [19]. When ionic surfactants are used, the particle’s charge will change according to the surfactant’s charge [19,20]. Due to this fact, it is, e.g., possible to test the impact that particles with different charges have on the uptake in cells [7]. 

The process allows using various biologically degradable polymers for the formulation of nanoparticles [9,21]. One of those commonly used polymers is polycaprolactone (PCL). With this polymer, nanoparticles can be formulated, which are stable in dispersion for a long period due to the polymers slow biodegradation [22].

Although this field of research has made significant progress, upscaling of the process is still an issue [3,23]. Nanoparticles formulated by the ouzo effect are often produced in a discontinuous process [13], with low polymer fractions of 1% [1], yielding dispersions with low solids contents [9]. Therefore, the quantity of dispersions as well as particles is always limited [3,7]. Furthermore, the proportion of organic to aqueous phase changes with every drop of organic phase that is released in the process, changing the environment for particle formation, and thus reducing the control over particle size [9]. To use those particles as carriers for active ingredients, it is desirable to create a continuous process, which can generate enough particles to make them economically viable. An answer to address the issue is the use of specifically designed mixers [4,9,24,25,26,27,28]. The organic phase and the aqueous phase are pumped in those mixers where the formulation of the particles takes place. Subsequently, the product is directed out of the mixer and can be obtained [9]. Usually, those mixers are T- or Y-shaped [9], but there are also other formations used for nano- or microcapsule production, where differently arranged channels can be seen [23,29,30]. Among other polymers [25], PCL has already been used in continuous nanoprecipitation processes [31,32,33], yielding dispersions with low solids contents [9]. Therefore, the quantity of dispersions as well as particles is always limited [3,7]. Furthermore, the proportion of organic to aqueous phase changes with every drop of organic phase that is released in the process, changing the environment for particle formation, and thus reducing the control over particle size [9]. To use those particles as carriers for active ingredients, it is desirable to create a continuous process, which can generate enough particles to make them economically viable. An answer to address the issue is the use of specifically designed mixers [4,9,24,25,26,27,28]. The organic phase and the aqueous phase are pumped in those mixers where the formulation of the particles takes place. Subsequently, the product is directed out of the mixer and can be obtained [9]. Usually, those mixers are T- or Y-shaped [9], but there are also other formations used for nano- or microcapsule production, where differently arranged channels can be seen [23,29,30]. Among other polymers [25], PCL has already been used in continuous nanoprecipitation processes [31,32,33].

A quick and convenient method to manufacture structures with small dimensions [34,35,36], such as the microfluidic models, is using one of the various 3D printing or additive manufacturing methods [37]. With such an easy-to-use and cost-efficient method, custom designed objects can be produced in a short period of time [38]. Among other methods, such as stereolithography (SLA), polyjet printing, or laser sintering, the fused deposition modeling (FDM) is one of the most widespread techniques when it comes to 3D printing technologies [39]. In this process, melted thermoplastics are extruded onto a building platform in the form of lines that one after the other complete the desired object [38,40]. FDM already offers simple production of customized printed parts from various commercially available and custom-made polymeric filaments. Several examples have shown the versatility of the technique in terms of materials and applications. Labware and mixing devices were fabricated from polypropylene and used to conduct a variety of reactions [41,42,43]. Moreover, Zhang et al. recently worked on the improvement of polyamide-based composites [38], and Wang et al. have given an introduction on how to fabricate thermoplastic functionally gradient composite parts [44]. Li et al. showed that even conducting polymers can be used as custom-made filaments for printing flexible electronic devices [45].

The main goal of this study is to establish a continuous process for nanoparticle formation using the polymeric ouzo effect through low-cost 3D-printed mixing devices. Several mixing devices with different geometries were printed with an FDM printer and used for the process. Different flow rates of the organic and aqueous phase were tested. Furthermore, different surfactants were used to control the particle’s stability and zeta potential. The results showed that the continuously produced nanoparticles tend to have larger diameters than the discontinuously formulated particles when using the same amount of organic and aqueous phase. Nevertheless, the results of using different flow rates showed that the particle’s size decreased upon increasing the flow rate of the organic and aqueous phases. The charge of the particle’s surface was successfully adjusted in a positive and negative region using ionic surfactants. Additionally, it was possible to create uncharged particle surfaces by using non-ionic surfactants. With the parameters used for the experiments, the particle sizes were almost independent of the mixing geometry.

## 2. Materials and Methods

### 2.1. Chemicals

Polycaprolacton (PCL, MW 14.000) and Nile red (NR, MW 318.37) were purchased from Sigma-Aldrich (St. Louis, MO, USA). Acetone, sodium lauryl sulfate (SDS) (99%—MW 288.38) and tween 80 (T80) were purchased from Carl Roth (Karlsruhe, Germany). Lutensol AT50 (LAT50) was a donation from BASF (Ludwigshafen, Germany). Cethyltrimethylammoniumbromide (CTAB) (99%—MW 364.46) was purchased from Merck (Darmstadt, Germany). Deionized water was used throughout the experiments. All chemicals were used as received.

### 2.2. Construction and Printing of Micromixers

**Figure 2:**
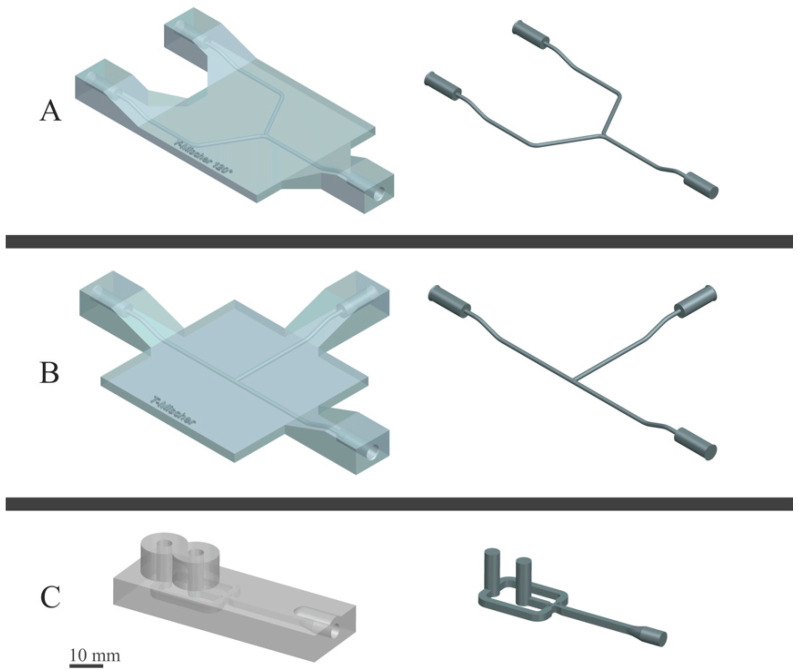
Schematic representation of the mixers used for the continuous nanoprecipitation and the orientation of their channels. (**A**) Y-mixer, (**B**) T-mixer and (**C**) focus-geometry micromixer.

The mixer models shown in Figure 2 were constructed with the software package Siemens NX^®^ (Siemens, München, Germany). The mixer models were setup as an assembly. Hence, the geometry of the mixer itself had to be replaced and all other components like external connections could be used again in all models. The .stl files were sliced with Ultimaker Cura (Utrecht, The Netherlands). Printing parameters are listed in the Table S1 (see Supplementary Materials). Ultimaker Nylon filament with a diameter of 2.85 mm was used on an Ultimaker 3 printer with an Ultimaker adhesive sheet on glass as print bed. The channel diameters of the mixers with Y and T geometry were 1.5 mm with circular cross section. The channels of the focus mixer were 2 mm reduced to 1 mm at the focusing point with square geometry. The entire set of mixer dimensions can be found in Figures S1–S3 (see Supplementary Materials).

The printed objects were used without further treatment. For connecting the mixers to the pumps, M6 screw threads were cut using a thread cutter.

### 2.3. Discontinuous Nanoparticle Formulation

Nanoparticles were formulated using the polymeric ouzo effect. In the discontinuous process, 4 mL of water were transferred into a sample vessel. Subsequently, 1 mL of the organic phase was added under constant stirring, starting the nanoprecipitation. The organic phase consisted of acetone and polycaprolactone. The concentrations of the dissolved polycaprolactone ranged from 0.01 wt% to 1 wt%. The amounts are listed in Table 1.

### 2.4. Continuous Nanoparticle Formulation

**Figure 3:**
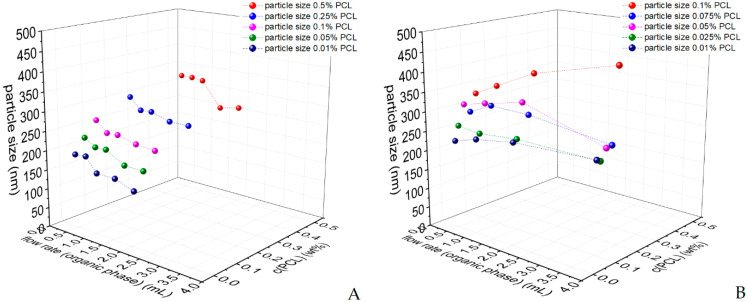
Particle sizes obtained by using different concentrations of PCL and varying flow rates in the continuous process (Y-mixer) (**A**) with a ratio o/w of 1:4, and (**B**) with a ratio o/w of 1:1.

For the continuous process, the organic phase and the aqueous phase reservoirs were connected to two hose pumps (Ismatec REGLO-CPF Digital, Wertheim, Germany). The polytetrafluorethylene tubes transporting the organic and aqueous phase were connected to a Y-shaped mixer, created with 3D-printing (see above) with standard 1.59 mm HPLC connectors. Polycaprolactone concentrations ranged from 0.01 wt% to 1 wt% (Figure 3). Different flow rates were used in a ratio of 1:1 and 1:4 of organic to aqueous phase (o/w). The effect of different mixers on the nanoparticles was tested using a T-shaped mixer in two different orientations and another mixer design [29]. Nile red was dissolved in the organic phase to show the influence of an encapsulation and to obtain fluorescently labelled nanoparticles. All experiments were conducted by triplicate. Additionally, attempts have been performed where the nonionic surfactant lutensol AT50 and Tween 80, and the ionic surfactant SDS and CTAB were dissolved in the aqueous phase to functionalize the particle’s surface. The concentrations of the surfactants in the aqueous phase were 0.5 wt% for the nonionic and 0.1 wt% for the ionic surfactant. The effect of surfactant dissolved in the organic phase was tested with 0.5–2 mg/mL.

### 2.5. Particles Size Analysis

The size of the particles was measured using Microtrac MRB’s NANO-flex (Haan, Germany). Approximately 50 µL of the dispersion was placed on the fiber optic window without dilution. Each sample was measured by triplicate. The particle sizes were given as volume distribution in integral and differential form. As a result, the particle sizes were given as a percentage distribution. The given data were put into a gauss curve with the program, OriginPro 2017G 64 bit (OriginLab. Northampton, MA, USA), in order to receive an average. Furthermore, the FWHM (full width half maximum) were used as measurements for the particle size distribution and denoted as variability *w*.

### 2.6. Zeta Potential

Zeta potential was measured using Stabino particle charge mapping. For the determination of the zeta potential, ten milliliters of the dispersion were filled in the provided sample vessel. Each sample was measured by triplicate.

### 2.7. Atomic Force Microscopy

AFM (Atomic force microscopy) was performed using Nanosurf Easyscan 2 (Liestal, Switzerland). For the sample preparation, 20 µL of the nanoparticle dispersion was left on a slide and dried at room temperature overnight. The slide with the dried sample was placed below the cantilever (Tap190Al-G) and a non-contact mode was started. An area of 625 µm^2^ was scanned with 128 lines. The time per line was set to 1 s.

## 3. Results and Discussion

### 3.1. Discontinuous Process for the Formulation of PCL Nanoparticles

The aim of this study was to clarify how the scale up of the nanoprecipitation to a continuous process affects the particle’s size. As reference for the particles created with the continuous process, dispersions were formulated with the discontinuous process described in other studies [9,13]. Therefore, for the discontinuous process, several concentrations of PCL (Table 1) and the ratio o/w of 1:4 were used. This ratio produced the most stable dispersions.

The results in Table 1 show that an increasing particle size was observed, increasing PCL concentration in acetone. Particle sizes from approx. 130 nm (0.01 wt%) to 260 nm (1 wt%) were obtained. As shown in Table 1 the particle’s size variability was not affected by the concentration of the polymer in the organic phase. By comparing the particle sizes with values from the literature [13], it exposed that the size of the particles generated in this study is about 100 nm larger than the size of the nanoparticles formulated by Badri et al.; however, the particle size is in the same range, well below 500 nm. Two major differences may explain this deviation. Firstly, Badri et al. used a rotary evaporator to evaporate the organic phase from the product and they made the particles with a ratio o/w of 1:2. Here, the acetone evaporated at room temperature, which is, according to Badri et al., a factor that creates larger particles. Secondly, we used a ratio o/w of 1:4 because this ratio produced the most stable dispersions, and dispersions created with a ratio o/w of 1:2 showed increased particle sizes. The results showed that particles with a concentration of 0.5 wt% PCL are stable for 2 weeks, while particles created with 0.1 wt% PCL were stable for more than a month, when those particles were produced with a ratio o/w of 1:4. Particles with a concentration of 1 wt% were stable for one day. After the given time, the particles coagulated to macroscopically visible aggregates.

### 3.2. Continuous Process for the Formulation of PCL Nanoparticles

Based on the reference dispersions produced by a discontinuous process (Table 1), dispersions were formulated with a continuous process (Y-mixer) with the equal ratio of organic to aqueous phase (1:4) and equal concentrations of PCL. Different flow rates have been used to test the impact of the flow rates on the particle’s size. Subsequently tests with a ratio o/w of 1:1 have been performed to get an overview on how different volumes of water impact the formulation besides the concentration of PCL dissolved in the organic phase.

Figure 3A shows a 3D graph of the particle’s sizes formulated with a ratio o/w of 1:4 and varying concentrations of PCL from 0.5 wt% to 0.01 wt%. Figure 3 shows the correlation of the particle size on the concentration of PCL dissolved in the organic phase and on the flow rate was visualized. Compared to the results of the discontinuous process, the particles were smaller, when a lower amount of PCL was used for the formulation. This fact was observed in all different flow rates as well. Looking at the effect different flow rates have on the particle’s size one can see that the size decreases upon increasing the flow rate. Within all concentrations of PCL, the particle size decreased by a factor of 15 to 25% upon increasing the flow rates from 0.4:1.6 to 2:8 mL/min (o/w). This outcome correlates with the findings ported by other studies [9]. Overall, the results showed that the particle’s size could be adjusted in a range from 130 to 360 nm using different concentrations of PCL and varying flow rates.

Figure 3B shows the results of the particle sizes formulated with an o/w of 1:1 and differing concentrations of PCL from 0.1 wt% to 0.01 wt%. Comparing the results shown in Figure 3B with the ones in Figure 3A, some difference appears. First, the fact that increasing concentrations of PCL dissolved in the organic phase led to increasing particle sizes is observable in Figure 3B. Additionally, a flow rate of 4 mL:4 mL showed that this rule was not visible in all flow rates. Second, the effect of different flow rates on the particle sizes differed from those seen in Figure 3A and others reported [9]. While increasing the flow rate from 1 mL:1 mL to 4 mL:4 mL, it shows that the particle size increased using concentrations of 0.1 and 0.01 wt% PCL. The experiments with the concentration 0.025 wt% show more or less constant values and the concentrations 0.05 and 0.075 wt% initially yield particles with increasing and later decreasing size. This contradiction might be traced back to the higher amount of organic phase, but it can also occur due to the lower stability of the dispersions, when using a ratio o/w of 1:1. In that case, particle enlargement might occur due to Ostwald ripening (higher solubility of the polymer in the continuous phase due to higher concentration of organic solvent) or due to aggregation of the particles (reduced colloidal stability).

Comparing the two graphs (Figure 3A,B), the effect of different volumes of water during the formulation can be seen. The particle sizes obtained with a ratio o/w of 1:1 can be found in a range between 220 nm and 465 nm, while the sizes obtained with a ratio o/w of 1:4 can be found in the range of 130 nm to 320 nm. The results clearly show that particles created with a lower amount of water (1:1) tend to be bigger than particles created with four times the amount of water (1:4). This effect is especially pronounced when comparing the particles created with 0.1 wt% PCL dissolved in the organic phase. Particles created with 0.1 wt% and a ratio o/w of 1:1 could be adjusted in a range of 330 to 465 nm when using different flow rates of the organic phase from 0.4 mL/min to 2 mL/min. Particles created with a ratio o/w of 1:4 could be adjusted in a range of 225 to 260 nm. Therefore, particles created with a higher amount of water are 30 to 45% smaller than the particles created with a lower amount of water, similar to the findings of another study [13]. Another factor that might explain this circumstance is, according to the literature, the mixing behavior, which is influenced by the flow rates [33]. Lince et al. discovered that the turbulence of the aqueous phase affects the mixing efficiency of the organic and the aqueous phase. Therefore, supersaturation can be increased by increasing the flow rates with unchanged diameter of the feeding tubes, leading to a decrease in particle size [33]. With the flow rates of the aqueous and the organic phase and the channel diameters, the Reynolds numbers were estimated to be in the range between 6 (water, 0.4 mL/min) and 140 (organic phase, 8 mL/min), which are both in the range of a laminar flow regime. Changes in flow regimes might be an additional reason why the particle size is decreasing when using a ratio o/w of 1:4 instead of 1:1; however, this remains a subject of further studies on the mixing behavior.

At last, the continuously produced particles need to be compared to the discontinuously produced particles. Considering the results given in Figure 3A and comparing them to the results given in Table 1, it is seen that the particle sizes created with a flow rate of 2 mL:8 mL presented the most similar behavior to the discontinuous ones.

**Figure 4:**
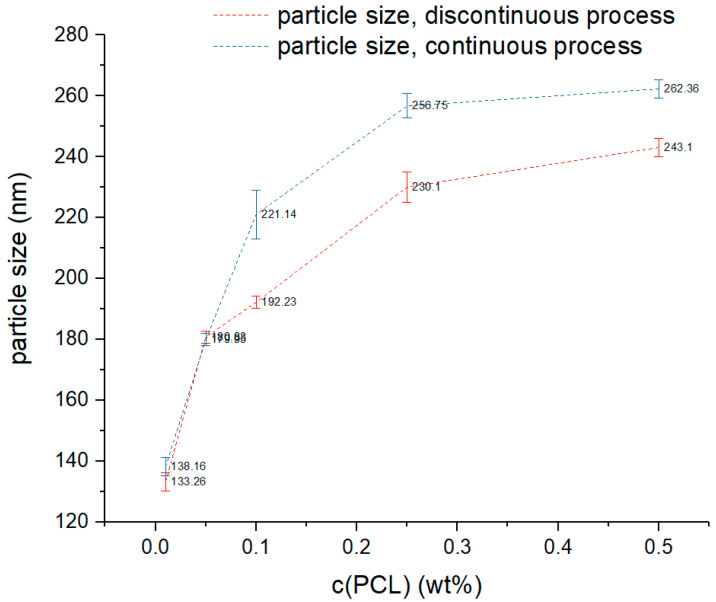
Particle sizes produced by a continuous process (Y-mixer) with a flow rate of 2 mL/8 mL compared to the particle sizes produced by a discontinuous process.

Table 2 shows the results of the continuously produced particles with a ratio o/w of 1:4 and a flow rate of 2 mL:8 mL. Figure 4 compares the results shown in Table 2 to the results of the discontinuously formulated particles shown in Table 1. The variability of the particle’s size created on a continuous process differs from the ones created on a discontinuous process, since the variability increases upon enlarging the concentration of PCL dissolved in the organic phase. The data shows (Figure 4) that the particles created in the discontinuous process are almost identical in size as the continuously produced particles when using concentrations of 0.01 wt% to 0.05 wt% PCL. The particles formulated with 0.1 wt% to 0.5 wt% PCL show size differences of 10% to 15%, implying that the deviation of the particle’s sizes increase upon rising the concentration of PCL. Overall, the data shows, that the size of continuously generated particles can also be adjusted in a characteristic range.

To summarize, the particle size is clearly dependent on the concentration of the polymer in the organic phase and dependent on the amount of water during the formation, for dispersions formulated with the discontinuous as well as the continuous process. Additionally, the flow rate adds a new factor that can be used to increase the control over the particle’s size using the continuous process.

### 3.3. Effect of Solvent Evaporation

Evaporation of the organic solvent is a crucial step to reach stability [9] and non-toxic dispersions [4]. Usually, the organic solvents (Acetone, THF, Ethanol) used in the nanoprecipitation method are toxic to the cells, making it inevitable to evaporate those solvents to get a non-toxic aqueous dispersion of nanoparticles [4]. According to Lepeltier et al., removal of the solvent is also reducing Ostwald ripening, as the solubility of polymer in the continuous phase is reduced, leading in an improvement of stability and constant particle size [9]. In some cases, particle dispersions are additionally diluted in water post-formulation to quickly decrease the effect of Ostwald ripening [27,33] by reducing the solubility of the polymer in the continuous phase as low as possible [46]. Here, experiments have been performed with an organic to aqueous phase ratio and flow rate of 1:4. The formulated nanoparticle dispersions were left open for 24 h to evaporate the acetone at room temperature. Nanoparticle’s size was measured immediately post formulation and after 24 h of evaporation at room temperature.

The results represented in Table 3 show that the particle sizes decrease by approx. 20 to 30 nm in each experiment. As expected, the particles are swollen with acetone, thus the size decrease, when the solvent is evaporated. No change in stability or any precipitates have been observed during at least one month Thus, we assume, that the solubility of the polymer in the continuous phase is low enough to prevent significant contribution of Ostwald Ripening on particle growth and the particles are in a quenched state directly after formation. Overall, the results show that particles can be created for cellular tests without having to consider the impact evaporation of the solvent has on the particles.

### 3.4. Effect of Surfactant

Several examples can be found in the literature, that long-term stable dispersions can readily be formulated with the polymeric ouzo effect [1,9,11,14]. In some cases, however, the use of surfactants may be beneficial. First, additional stability against coagulation and eventually phase separation is generated, and secondly, the particle surface charge can be controlled. As the phase separation is a fast process [11] in contrast to surfactant diffusion [47], non-ionic surfactants can screen the surface charge of the polymeric particles upon adhesion, shifting the zeta potential to the range of ζ = 0 mV [20,22]. Therefore, the surface charge can be adjusted by the use of surfactant. This functionalization might be useful when it comes to the effect of different zeta potentials on cellular uptake [8]. Thus, formulations with the non-ionic surfactants Tween 80 and Lutensol AT50, as well as the ionic surfactants SDS and CTAB, were prepared. Additionally, surfactant was added post-formulation to check if a functionalization of the particle’s surface can be done with completed particles.

The results of the particles produced with the surfactant in the organic phase are shown in Table 4. Particles created with the non-ionic surfactant LAT50 and T80 in the organic phase show minor changes in terms of size as particles formulated without surfactant.

Table 5 shows the particle sizes, the variability and the zeta potential of the particles prepared in the presence of surfactants. The particle size is not affected, when the non-ionic surfactant LAT50 was present. Formulations with other surfactants show a slight increase in particle size and variability, which may be attributed by an increased hydration shell, resulting from the adsorbed surfactant.

In addition, the values of the zeta potentials are shown in Table 5. Particles created without surfactants possess a slightly negative potential of ζ = −14 mV, which can be attributed to the carboxy-groups of the polymer PCL placed on the surface of the particles [48], resulting from partial hydrolysis or a terminal carboxy function [49]. This negative value decreases up on using the non-ionic surfactant LAT50 and T80 to a value of +8 (T80) or −5 (LAT50) since the surfactants adhere to the outer shell of the particle’s surface screening the negative charge of the carboxy-groups. If the ionic surfactants SDS and CTAB are solved in the aqueous phase, then the potential is shifted to a negative value of ζ = −62 mV or to a positive value of ζ = 40 mV. Results show that the surface charge of the particles can be adjusted with the appropriate surfactant.

The last two entries in Table 5 showed that the particle size as well as the zeta potential were changed to comparable values when CTAB or SDS was added to the dispersion post formulation, offering further possibilities to control surface properties. No change in dispersion stability could be observed, when comparing dispersions formulated with or without surfactant, independent of the other preparation condition, such as flow rate or polymer concentration.

To summarize, the particle charge can be controlled by the use of appropriate surfactant, to create differently charged particles for cellular uptake experiments.

### 3.5. Encapsulation of Fluorescence Dye Nile Red

Encapsulation of fluorescent dyes into the PCL particles enables tracking the uptake of those particles in a cell culture test. Therefore, encapsulation of the fluorescent dyes should be possible without any change in stability and morphology of the particles. To examine the effect encapsulation has on the particles, tests have been performed where 50 ng/mL Nile red was encapsulated in the particles. The results of those tests are shown in Table 6.

Particles created with 50 ng/mL of the fluorescent dye Nile red in the organic phase show no change in terms of size. Measurement of the zeta potential showed a value of ζ = −8 mV, being in a comparable area to particles created without Nile red. No loss of stability has been detected, since particles created with Nile red were stable for over a month as well. This implies that encapsulation of Nile red has no major impact on the process of formulation. Thus, the parameter established in the experiments shown above can be transferred to the formulation with Nile red. In summary, the results show that the effect of the encapsulation of Nile red can be neglected when it comes to formulating particles for the cause of cell culture tests.

### 3.6. Role of the Micromixer

Many studies have been performed regarding the optimal mixing device and orientation of the mixing channels. Most of them use a confined impingement jet (CIJ)-Mixer, which usually has a T- or Y-shaped design [9]. In this study, Y- and T-shaped mixers have been tested to see whether there is a difference in particle size resulting from using different geometries. The T-mixer has been used in two different orientations, one orientation where the steams meet at 180° and one where they meet in an angle of 90°. Furthermore, another mixing design (focus geometry) has been tested to see the impact of different mixing formations. The results of those tests are shown in Table 7.

The findings of this study show that the geometry of the mixer channels has no major impact on the particles size. Thus, it was identified that the nanoparticle formation with PCL, as the capsule forming polymer, is a robust process, with low dependency on the variation of geometry of the mixing channels.

### 3.7. Atomic Force Microscopy

**Figure 5:**
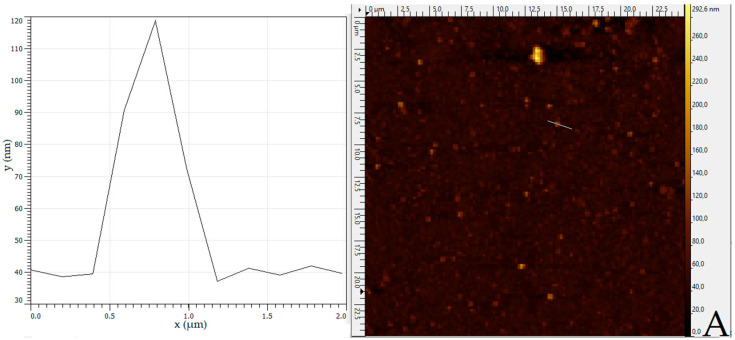

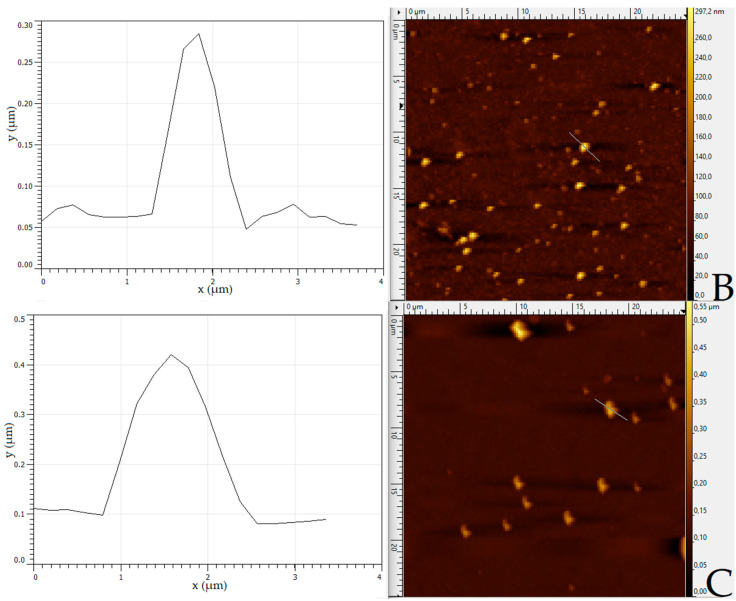
AFM image of particles created with (**A**) 0.01 wt% PCL, (**B**) 0.1 wt% PCL, and (**C**) 1 wt% PCL in the organic phase. The height image (left column) shows the diameter and height of representative particles.

The further use of nanoparticles in cell culture tests requires a full understanding of the particle’s properties. One factor that might be interesting when it comes to cellular uptake—besides the surface charge or particle size—is the morphology of the nanoparticles. Therefore, AFM was used to visualize particles created with a flow rate of o/a phase of 1:4 and varying concentrations of PCL in the organic phase from 0.01 to 1 wt%. The results of the AFM imaging are shown in Figure 5.

The results showed that the particle size increased with a higher concentration of the polymer in the organic phase, which can be seen in the height of the particles in Figure 5A–C. Furthermore, the sizes of the particles were in the expected range matching the results of Figure 3A. A closer look at Figure 5A–C also shows that the PCL dispersions were not clearly monodisperse. The nanoparticles produced generally presented size differences from 100 to 200 nm. At last, the results also showed that the deviation in particle size increased with the concentration of the polymer in the organic phase.

The morphology of the particles seen in Figure 5A,B is round, whereas the particles created with an amount of 1 wt% PCL in the organic phase (Figure 5C) showed an unsteady shape. This circumstance might be linked to the lower stability that has been discovered in this study. Overall, the results of the AFM imaging matched the findings in Section 3.1 and Section 3.2, that particle size is dependent on the initial concentration of the polymer.

## 4. Conclusions

In this study, we showed that the upscaling of polycaprolactone nanoparticle formulation to a continuous process can be accomplished using a self-printed CIJ-mixer. The results implicate that the decisive parameters affecting the size of the formulated particles of the discontinuous process can be transferred to the continuous one. Furthermore, new parameters, such as the flow rate of the phases, can be used to gain even more control over the particle’s size and solids content. Attempts, with three different mixing designs and four different geometries, proved, that the continuous nanoprecipitation with PCL is a robust process, which is not highly affected by the orientation of the channels in which the two phases are led into each other.

Functionalization of the particle surface via surfactant showed that the particle’s surface charge can not only be adjusted during formulation, but can also be done with preformulated particle dispersions. Due to this fact, it is possible to create differently charged particles made of the same dispersion increasing the reproducibility when it comes to comparing the effect of the different surfactants on particles with identical size and size distribution. Finally, we showed that polycaprolactone is an easy-to-use polymer for the nanoprecipitation due to the particles being stable for a long period without the use of surfactant.

## Figures and Tables

**Table 1:** Particle sizes achieved by a discontinuous process using different concentrations of PCL.

PCL Concentration (wt%)	Particle Size, Discontinuous Process (nm)	Variability (nm)
0.01	133 ± 3	73 ± 9
0.05	180 ± 2	90 ± 7
0.10	192 ± 2	84 ± 5
0.25	230 ± 5	71 ± 10
0.50	243 ± 3	94 ± 8
1.00	262 ± 4	95 ± 10

**Table 2:** Particle sizes produced by a continuous process (Y-mixer—flow rate 2 mL:8 mL).

PCL Concentration (wt%)	Particle Size Continuous Process (nm)	Variability (nm)
0.01	138 ± 3	62 ± 8
0.05	179 ± 2	58 ± 5
0.10	221 ± 8	109 ± 19
0.25	256 ± 4	102 ± 9
0.50	262 ± 3	101 ± 8

**Table 3:** Difference in particle sizes before and after evaporation of solvent.

Concentration of PCL (wt%)	Particle Size with Acetone (nm)	Particle Size after Evaporation (nm)	Difference in Particle Size (nm)
0.01	155 ± 1	119 ± 2	36
0.05	208 ± 2	183 ± 3	25
0.10	236 ± 3	206 ± 3	30
0.25	271 ± 3	252 ± 4	19
0.50	318 ± 5	299 ± 5	19

**Table 4:** Influence of the concentration of nonionic surfactants dissolved in the organic phase (Y-mixer).

Configuration	Particle Size (nm)	Variability of the Particle Sizes (nm)
0.1% PCL	236 ± 3	88 ± 6
0.5 mg LAT50	215 ± 8	77 ± 18
1 mg LAT50	268 ± 4	136 ± 12
2 mg LAT50	260 ± 3	132 ± 8
0.5 mg T80	293 ± 5	165 ± 12
1 mg T80	276 ± 3	110 ± 8
2 mg T80	312 ± 4	178 ± 10

**Table 5:** Particle sizes and zeta potential of particles prepared in the presence of surfactants.

Configuration	Particle Size (nm)	Variability of the Particle Sizes (nm)	Zeta Potential (mV)
0.1% PCL	236 ± 3	88 ± 6	−14
0.5% LAT50 in aq.	246 ± 6	111 ± 16	−5
0.5% T80 in aq.	279 ± 3	141 ± 7	8
0.1% SDS in aq.	262 ± 2	127 ± 4	−62
0.1% CTAB in aq.	252 ± 2	128 ± 5	+41
0.1% SDS post formulation.	271 ± 4	125 ± 9	−51
0.1% CTAB post formulation.	281 ± 6	168 ± 14	+51

**Table 6:** Particle sizes of dispersions prepared with or without Nile red (Y-mixer).

Polymer Concentration (wt%)	Particle Size (nm)	Variability of the Particle Sizes (nm)
0.01%	155 ± 1	55 ± 2
0.05%	208 ± 2	89 ± 6
0.1%	246 ± 3	88 ± 6
0.01% + NR	159 ± 3	64 ± 6
0.05% + NR	208 ± 2	90 ± 6
0.1% + NR	219 ± 3	86 ± 6

**Table 7:** Particle sizes obtained using different mixers.

Attempt	Particle Size (nm)	Variability of the Particle Sizes (nm)
0.1% PCL T-Micromixer 0.1% PCL T2-Micromixer	223 ± 3 224 ± 2	87 ± 7 80 ± 5
0.1% PCL Y-Micromixer	236 ± 3	88 ± 6
0.1% PCL focus geometry	229 ± 4	79 ± 11

## Data Availability

All data are contained within the article.

## Supplementary Materials

The following supporting information can be downloaded at: https://www.mdpi.com/article/10.3390/polym14081509/s1, Section 1: Printing Parameters, Figure S1: Dimension of the mixer with T-geometry; Figure S2: Dimension of the mixer with Y-geometry; Figure S3: Dimensions of the mixer with focus geometry; Table S1: Printing parameters.
