# Supplementary material for: Continuous Nanoprecipitation of Polycaprolactone in Additively Manufactured Micromixers
Source: Polymers (Basel). 2022 Apr 7;14(8):1509. doi: 10.3390/polym14081509 (PMC9032806; doi:10.3390/polym14081509)
Supplement: Supplementary file 1 [file polymers-14-01509-s001.zip › polymers-1617664-supplementary.pdf]

polymers MDPI

Supplementary Materials

Continuous Nanoprecipitation of Polycaprolactone in Additively Manufactured Micromixers

Simeon Göttert [1], Irina Salomatov [1], Stephan Eder [1], Bernhard C. Seyfang [1], Diana C. Sotelo [2], Johann F. Osma [2] and Clemens K. Weiss [1,*]

[1] Technische Hochschule Bingen, Life Sciences and Engineering, Berlinstrasse 109, 55411 Bingen, Germany; simeon.goettert@stud.th-bingen.de (S.G.); irina.salomatov@web.de (I.S.); s.eder@th-bingen.de (S.E.); b.seyfang@th-bingen.de (B.C.S.)
[2] Department of Electrical and Electronic Engineering, Universidad de los Andes, Cra. 1E No. 19A-40, Bogotá 111711, Colombia; dc.sotelo10@uniandes.edu.co (D.C.S.); jf.osma43@uniandes.edu.co (J.F.O.)
* Correspondence: c.weiss@th-bingen.de; Tel.: +49-6721-409270

1. Printing Parameters

All models were printed on an Ultimaker3 with Ultimaker transparent Nylon filament with a diameter of 2.85 mm. The .stl files obtained from the CAD construction software was imported to Cura 4.x for slicing. The Ultimaker advanced printing kit adhesive sheet was used to increase adhesion to the build plate. The printing parameters were identical for all of the models.

Table S1. Printing parameters.

Parameter	Value
Layer Height	0.1 mm
Wall Thickness	1.3 mm
Wall Line Count	3
Top/Bottom Thickness	1.2 mm
Top/Bottom Line Count	12
Infill Density	50%
Infill Pattern	Gyroid
Printing Temperature	245.0 °C
Build Plate Temperature	85 °C
Printing Speed	70.0 mm/s

2. Model Dimensions

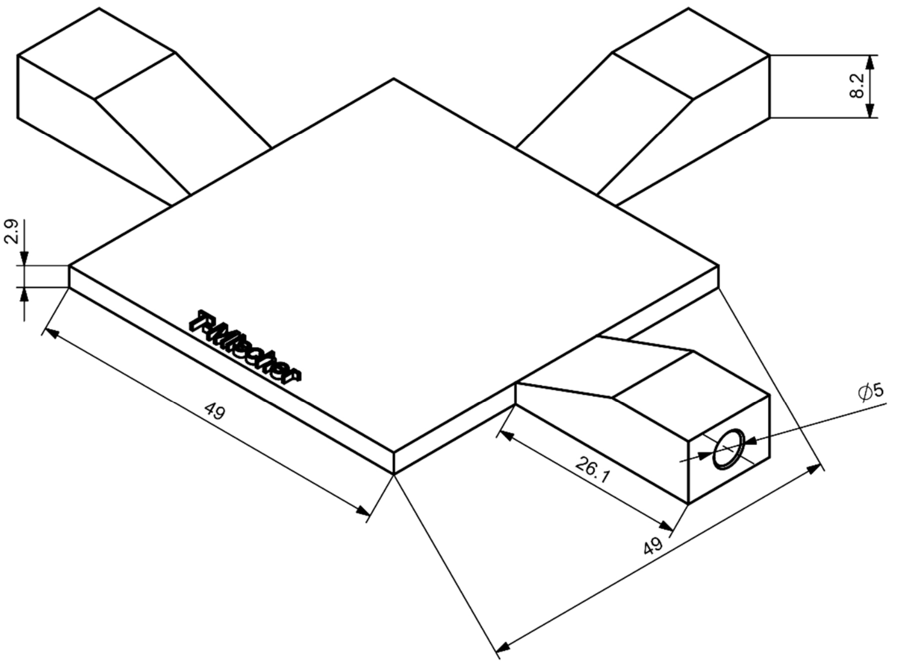

Figure S1. Dimensions (mm) of the mixer with T-geometry.

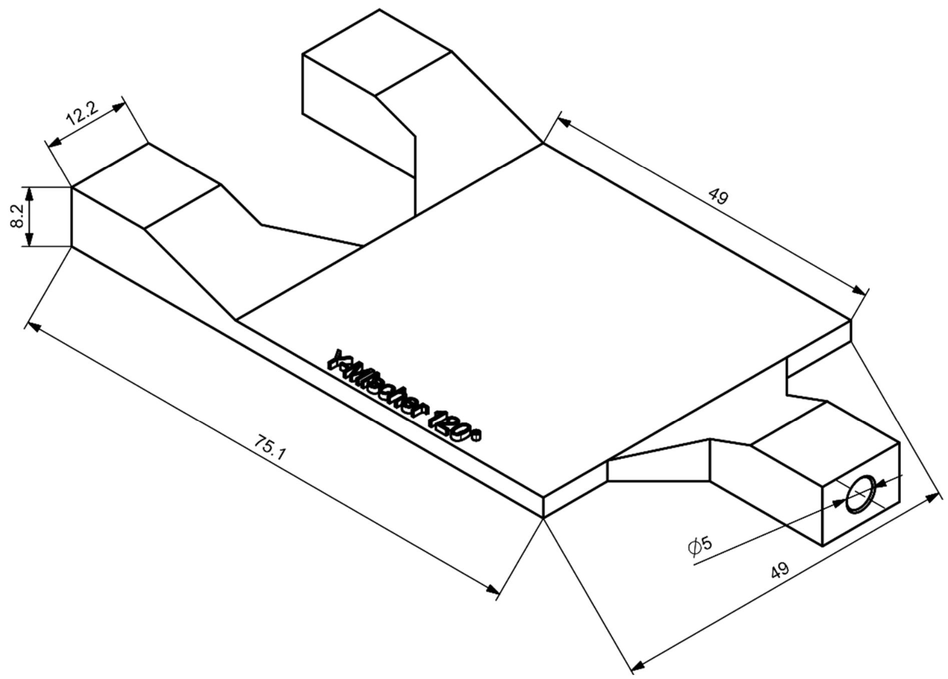

Figure S2. Dimensions (mm) of the mixer with Y-geometry.

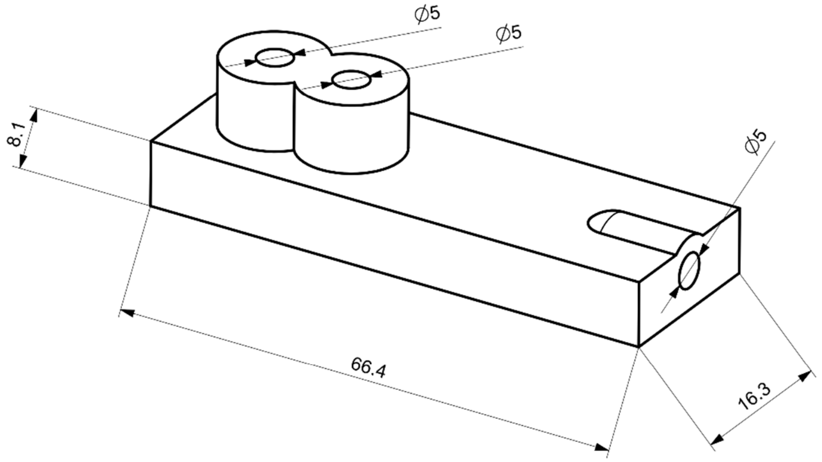

Figure S3. Dimensions (mm) of the mixer with focus geometry.
